# Multiomics analysis profile acute liver injury module clusters to compare the therapeutic efficacy of bifendate and muaddil sapra

**DOI:** 10.1038/s41598-019-40356-5

**Published:** 2019-03-13

**Authors:** Ainiwaer Talifu, Refuhati Saimaiti, Yusufu Maitinuer, Geyu Liu, Miernisha Abudureyimu, Xuelei Xin

**Affiliations:** 1 State Key Laboratory Basis of Xinjiang Indigenous Medicinal Plants Resource Utilization and The Key Laboratory of Plant Resources and Chemistry of Arid Zone, Xinjiang Technical Institute of Physics and Chemistry, Chinese Academy of Sciences, Urumqi, 830011 China; 2 Hospital of Xinjiang Traditional Uighur Medicine, Urumqi, 830001 China; 3 University of Chinese Academy of Sciences, Beijing, 100039 China

## Abstract

The pathogenesis of acute liver injury has been plagued by biologists and physicians. We know little about its therapeutic mechanism. Therefore, this study explored the mechanism of bifendate and muaddil sapra in the treatment of acute liver injury. Firstly, co-expression and cluster analysis of disease-related genes were carried out, and the Go function and KEGG pathway of modules and related genes were identified. Secondly, pivot analysis of modules can identify key regulators. On the other hand, based on the acute liver injury induced by CCl4, we use the combined analysis of proteomics and transcriptome to find therapeutic targets and related mechanisms of drugs. A total of 21 dysfunction modules were obtained, which were significantly involved in immune system, hepatitis and other related functions and pathways. Transcriptome analysis showed 117 targets for bifendate treatment, while 119 for muaddil sapra. Through exploring the mechanism, we found that the two drugs could modulate the module genes. Moreover, bifendate regulate the dysfunction module through ncRNA (SNORD43 and RNU11). Muaddil sapra can mediate dysfunction modules not only by regulating ncRNA (PRIM2 and PIP5K1B), but also by regulating TF (STAT1 and IRF8), thus having a wider therapeutic potential. On the other hand, proteome analysis showed that bifendate mainly regulated Rac2, Fermt3 and Plg, while muaddil sapra mainly regulated Sqle and Stat1. In addition, muaddil sapra regulates less metabolic related proteins to make them more effective. Overall, this study not only provides basic theory for further study of the complex pathogenesis of acute liver injury, but also provides valuable reference for clinical use of bifendate and muaddil sapra in the treatment of acute liver injury.

## Introduction

With the continuous improvement of living environment and medical level, therapies applied to acute liver injury have emerged in an endless stream. Such methods as non-surgical treatment management^1–3^ have greatly improved the survival rate of patients. However, its unknown pathogenesis and lethal factors remind us all the time that acute liver injury still threatens the health of humans worldwide. Acute liver injury has long attracted the attention of biologists and medical scientists, and many of the experimental and research results of acute liver injury related genes are included in the National Center for Biotechnology Information (NCBI-Gene) database.

For example, a series of studies have shown that in many species, biochemical enzymes such as alanine aminotransferase [ALT] and alkaline phosphatase [ALP] activity rise high are inseparable with liver damage and regeneration^4^. Corrick RM *et al*.’s study showed that the liver growth hormone (hGH) showed a resistance effect after acute injury, and the GH induced signal transduction and transcription activator 5 phosphorylation significantly decreased after the completion of the trauma process^5^. On the other hand, acute liver injury is closely related to the regulation of the immune system. It has been confirmed that the down-regulation of MyD88 and the inhibition of NF-κB decrease the expression of inflammatory protein MIP-1α in phagocytic cells. The production of serum alanine transaminase and pro-inflammatory cytokines such as interferon-γ and tumor necrosis factor-α eventually induce acute liver injury^6^. Therefore, the targeted promotion of MyD88 expression and activation of the NF-κB pathway has become a new idea for drug development and diagnostic therapy. TLR4 ligand lipopolysaccharidecan activate the expression of tumor necrosis factor-alphaand interleukin (IL)-6, leading to necrosis of hepatocytes, causing fulminant hepatic failure^7^. After injury, liver-specific transcription factors HNF-1α and HNF-4α play an important regulatory role^8^. In addition, the extracellular matrix protein Nephronectin (Npnt) plays a key role in the development of the kidney. Its ectopic expression in the liver cells exacerbates the acute hepatitis and liver injury induced by Con A so that it is identified as a potential treatment target for acute and chronic hepatitis^9^. These important results have provided valuable guidance for this research work and have greatly inspired our thinking.

Although previous researchers have reported a series of research findings on acute liver injury, the overall effect of these results is still elusive. This work from a global perspective to observe co-expression modules and interactions of acute liver injury-related genes driven by TF and ncRNA. Systematic analysis can help us to fully understand the molecular mechanisms of acute liver injury. By investigating the molecular mechanisms of bifendate and muaddil sapra in the treatment of acute liver injury, we have further deepened our insights into its treatment mechanism and provided a valuable reference for clinical drug guidance.

## Results

### Acute liver injury related genes have significant co-expression in model mouse

**Figure 1:**
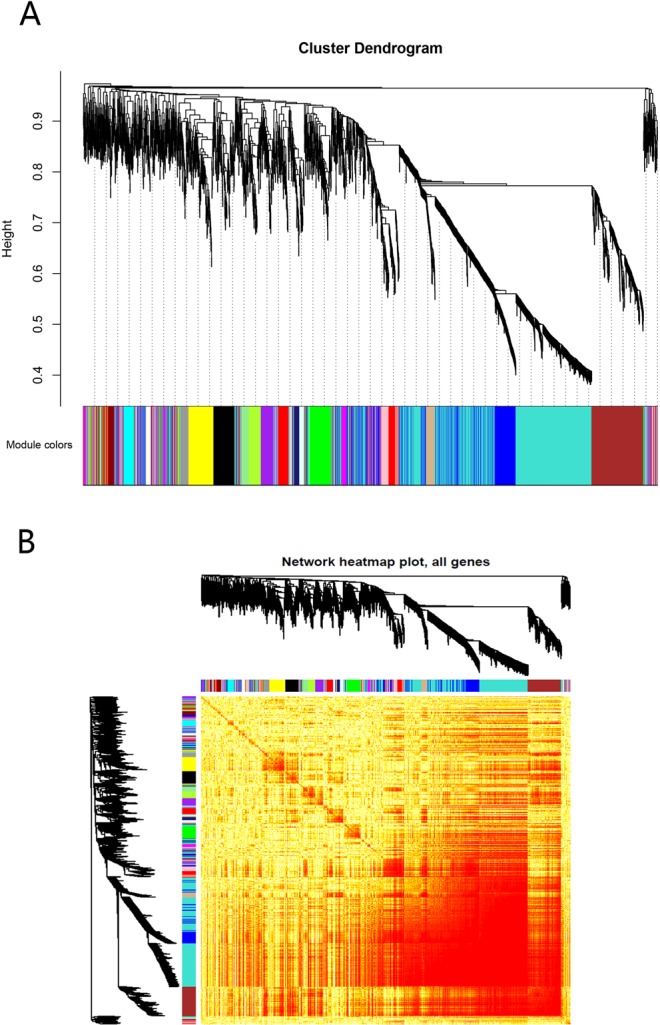
Co-expression modules.

To systematically study the mechanism of action of acute liver injury-related genes in mouse liver tissue treated with carbon tetrachloride (CCl4), we performed extensive data integration. We first constructed an expression matrix for 715 acute liver injury related genes and their interaction genes in model mouse liver tissue, including 4541 genes and 9 disease samples. Then, based on the weighted gene co-expression network analysis (WGCNA), we observed that these genes exhibited a significant group co-expression phenomenon in acute liver injury samples. The clustering of expression patterns of acute liver injury related genes in model mouse liver tissue into individual modules facilitates us to observe the complex cooperative relationships between these genes from the perspective of expression behavior. Therefore, by identifying the co-expression groups as the modules, we obtained 22 co-expression modules (Fig. 1A) that express genes with significant clustering in model mouse liver tissue (Fig. 1B). The core module with a block number of genes less than 500 was screened, and the last 21 co-expression modules were retained. The interaction between modules is the key to maintaining the relationship between the module and the global. It is also the key to exploring the function of the module.

**Figure 2:**
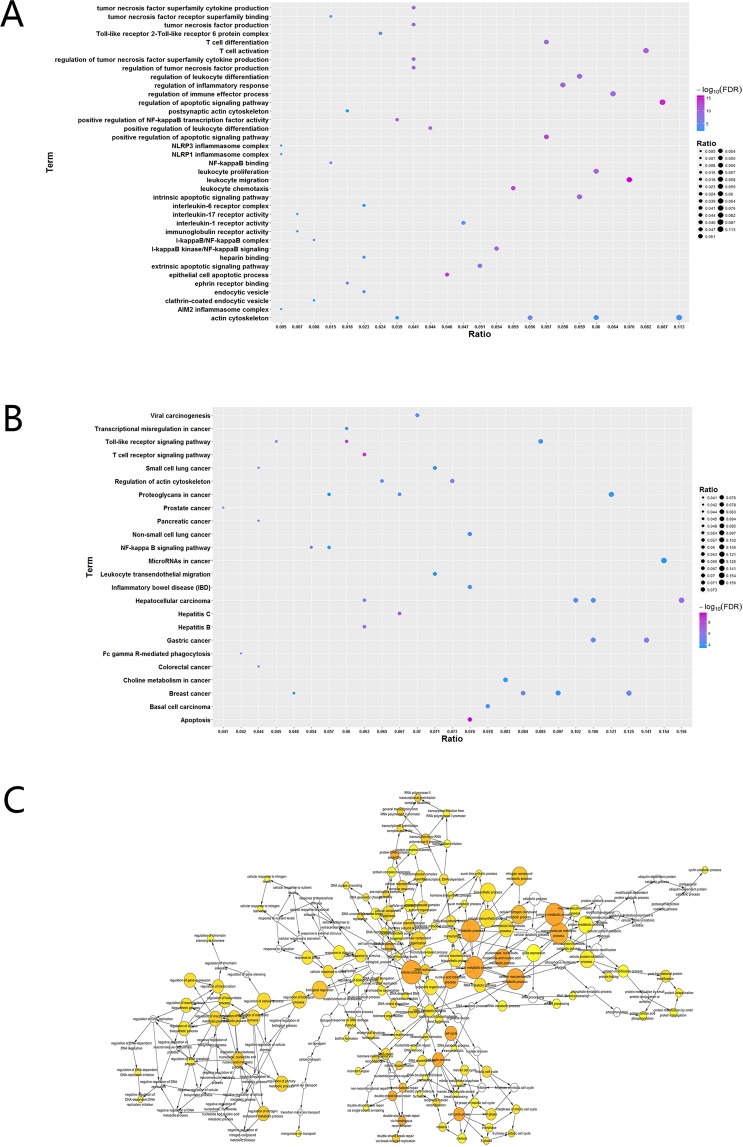
Enrichment analysis.

Subsequently, in the GO function analysis of 21 modules, a total of 614 cell composition entries, 260 molecular functional terms, and 9,971 biological processes were obtained. We found that these functions (Table S1, Fig. 2A) mainly focus on the regulation of immune regulation, inflammatory response, and regulation of cell carcinogenesis induced by injury. On the other hand, 21 modules were significantly enriched into 381 KEGG pathways (Table S2, Fig. 2B). In addition, statistical analysis of functions and pathways revealed that up to 16 modular genes were significantly involved in the positive regulation of cellular and cellular component movement. Another 14 modules significantly enriched the positive regulation of cell migration. Thirteen modules significantly involved in the regulation of systemic processes and regulation of protein serine/threonine kinase activity. Finally, the functional imbalance module is further analyzed from a global perspective by constructing a functional network (Fig. 2C).

### ncRNA and TF driving acute liver injury dysfunction modules

**Figure 3:**
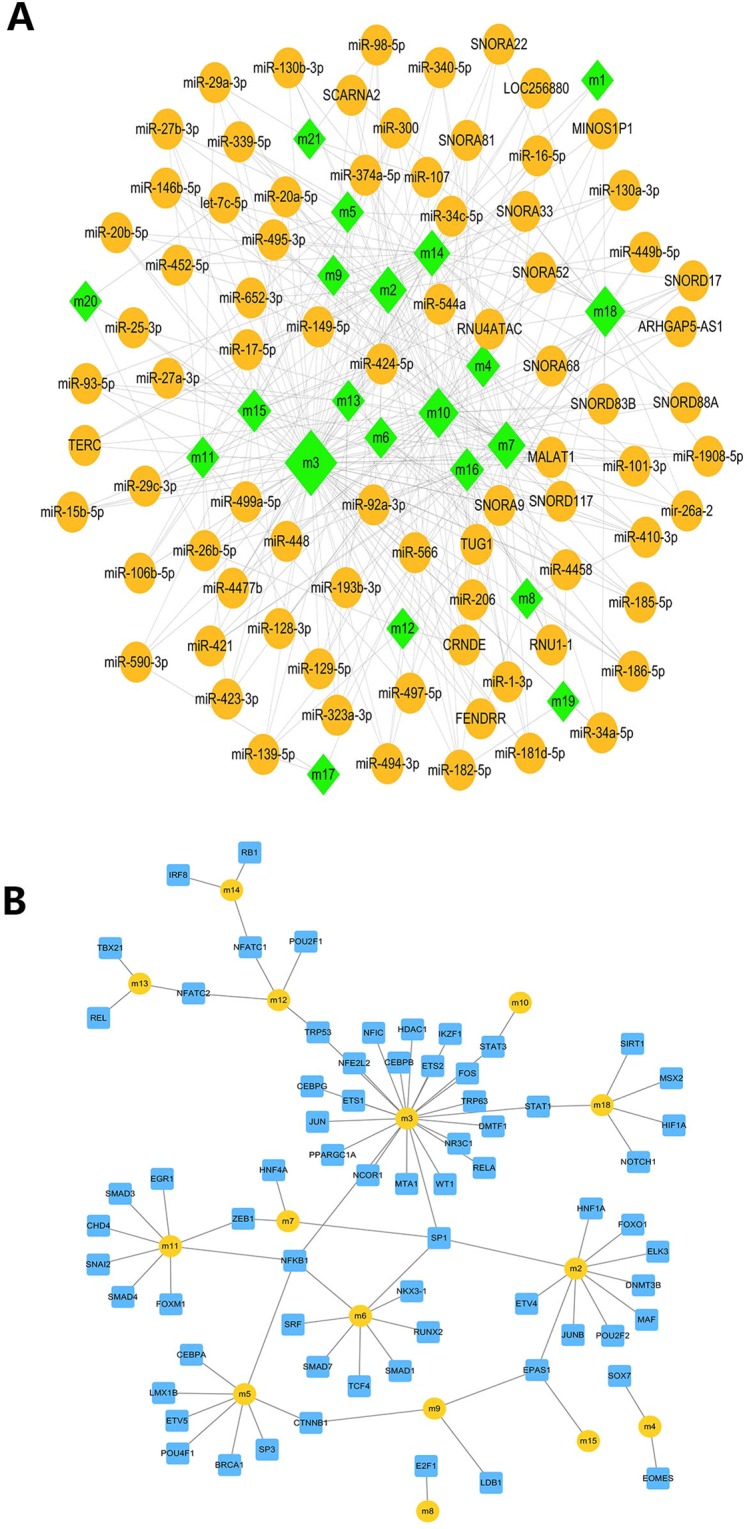
ncRNA and TF-pivot of regulatory module.

Dysfunction of modules is often accompanied by the regulation of non-coding RNA (ncRNA) and transcription factors. Therefore, we first predict and analyze the ncRNA pivot of the regulation module.2540 ncRNA-Module interaction pairs (Table S3, Fig. 3A),which involving 1550 ncRNAs. In the prominent pivots, long non-coding RNA (lncRNA) MALAT1 drives nine modules, while CRNDE and endogenous small RNA (miRNA) miR-26b-5p, miR-34c-5p, etc. drive seven modules. They even have a strong regulatory role in the overall situation, therefore identified as the core ncRNA.

In addition, in the prediction analysis of TF pivot, a total of 83 TF-Module control pairs involving 68 transcription factors were obtained (Table S4, Fig. 3B). Among them, NFKB1 mediates four modules, SP1 that mediates 4 modules and the EPAS1 transcription factor mediating 3 modules. These important TFs were identified as core transcription factors in the pathogenesis of acute liver injury.

### Identification of drug targets for bifendate and Muaddil sapra

**Figure 4:**
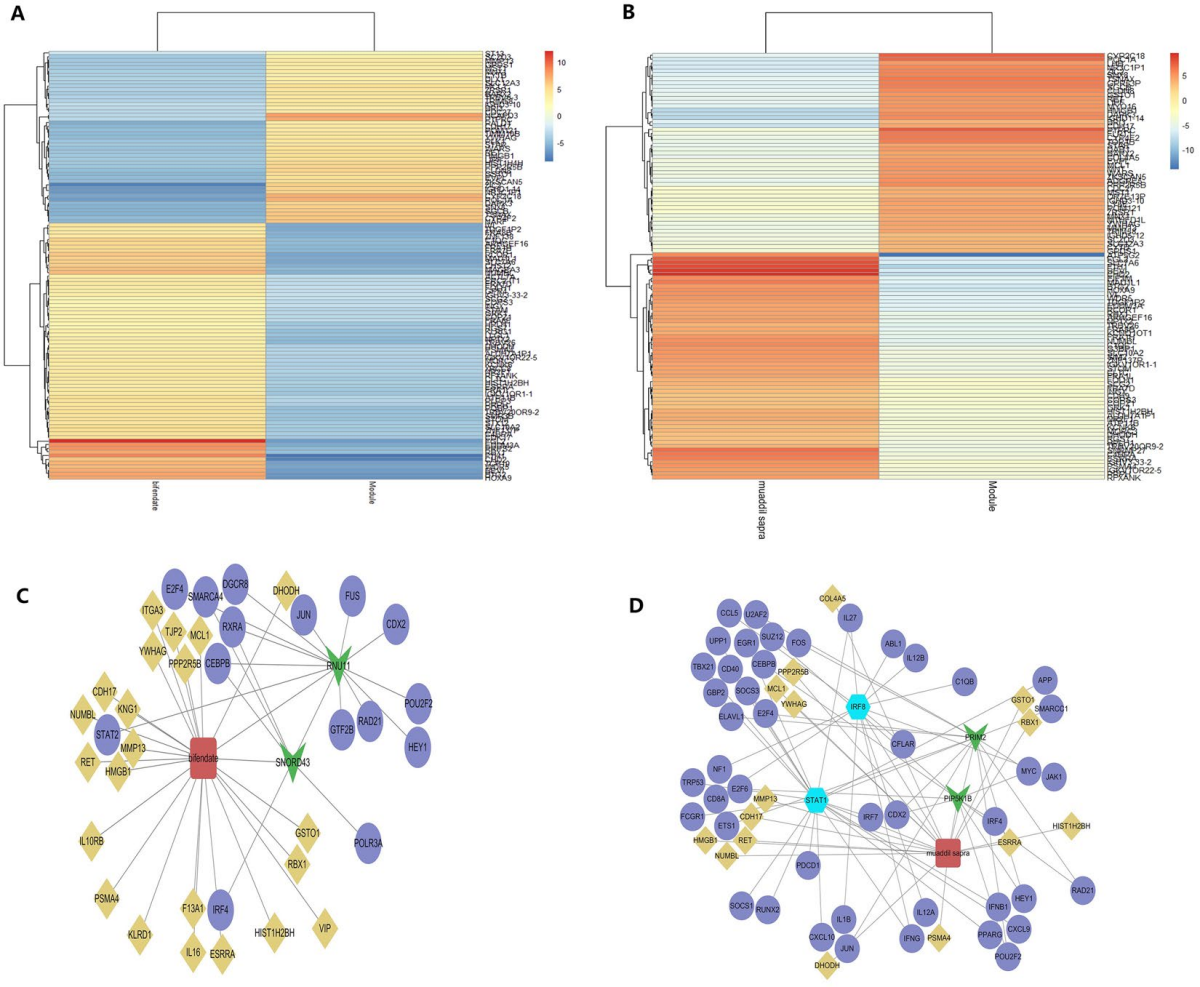
Identification of drug treatment targets.

Through transcriptome analysis of bifendate and muaddil sapra treated model mice, model mice and healthy control mice, the potential therapeutic mechanism of drugs can be further analyzed. Results there were 407 differential genes in the model group and the healthy group. There were 455 differentially expressed genes in bifendate group and model group. There were 440 differentially expressed genes in muaddil sapra group and model group. The differentially expressed genes after treatment were compared with disease related genes. We found that 47 of the genes up-regulated in the disease group were down-regulated after bifendate treatment, and we believe that they are targets for inhibition. 70 of the down regulated genes in the disease group were upregulated after bifendate treatment, and were considered as promote targets (Fig. 4A). Similarly, after muaddil sapra treatment, there were 51 inhibitory targets and 68 promotion targets (Table S5, Fig. 4B). Therefore, these genes can be considered as effective targets for drug therapy.

### Transcriptome analysis to compare the efficacy of bifendate and Muaddil sapra

Subsequently, we studied the mechanism of drug therapy. Twenty-two of the target genes of bifendate were found to exist in dysfunction modules and regulate eleven modules. Fifteen of the target genes of muaddil sapra also exist in the dysfunction module of acute liver injury in mice and regulate eight modules. It indicates that drugs can achieve therapeutic effect by targeting dysfunction modules.

On the other hand, the therapeutic gene of bifendate has the same genes as the ncRNA-pivot of the regulatory module, including SNORD43 and RNU11 (Fig. 4C). Muaddil sapra drug treatment gene has the same gene as TF-pivot, namely STAT1 and IRF8. In addition, the drug therapy gene of muaddil sapra has the same gene as ncRNA-pivot, including PRIM2 and PIP5K1B (Fig. 4D). It indicates that the two drugs can play a therapeutic role by regulating the module.

### Proteome analysis to compare the efficacy of bifendate and muaddil sapra

**Figure 5:**
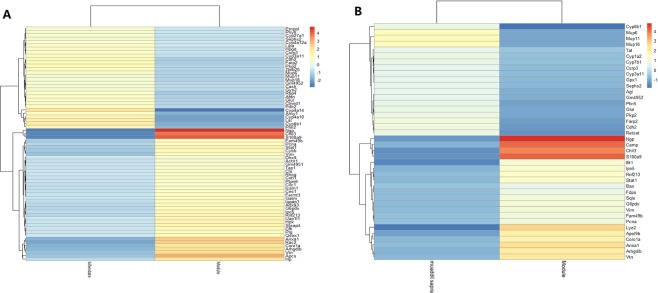
Target proteins of drugs.

Screening results of protein quantification between bifendate and muaddil sapra treatment group and acute liver injury model mice.142 (Table S6) and 92 (Table S7) differential proteins were obtained, respectively. Observing the functions and pathways involved in these proteins, it was found that both bifendate and muaddil sapra are involved in the regulation of T cell activation and other immune system processes. In addition, In addition, the differential proteins after drug treatment were compared with disease differential proteins. It was found that 39 target proteins were suppressed and 30 target proteins were promoted after bifendate treatment (Fig. 5A). After muaddil sapra treatment, there were 21 target proteins and 19 target proteins (Fig. 5B). Therefore, it is thought that drugs may play a therapeutic role by targeting these proteins.

**Figure 6:**
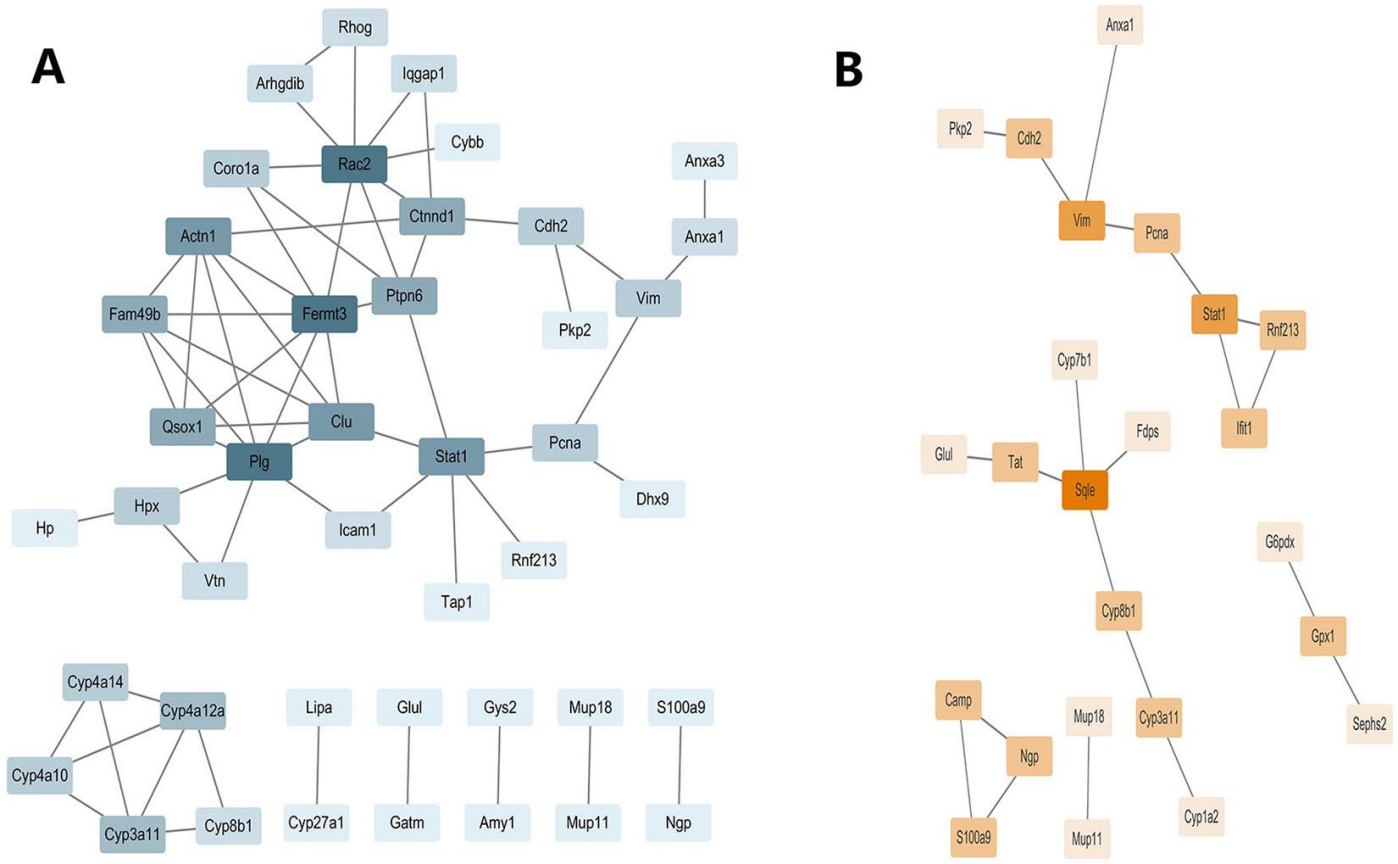
Interaction Network of Drug Target Proteins.

A protein interaction network was constructed for two differential proteins regulated by drugs. It was found that the therapeutic mechanism of bifendate is the interaction network cluster of Rac2, Fermt3and Plg as key molecules to regulate immunity and inflammation (Fig. 6A). The interactions of Cyp3a11, Cyp4a10 and Cyp4a14 network clusters serve as the main molecular mechanism of pharmacokinetics. On the other hand, in the treatment mechanism of muaddil sapra (Fig. 6B), Sqle and Stat1as the core protein modulates the modules, playing the main therapeutic effectiveness, while the interaction network cluster of Cyp1a2and Cyp3a11 is the main molecular mechanism of pharmacokinetics. From the point of view of therapeutic efficacy, bifendate not only activates the Stat1 regulatory modules, but also regulates the expression of Plg and Fermt3 to further regulate the immune system, eliminate inflammation, and play a rapid therapeutic role. From the pharmacokinetics of the two drugs, bifendate activates more metabolic molecules and has faster metabolic efficiency. In other words, the treatment of bifendate is great effective and metabolism is rapid, while the curative effect of muaddil sapra is milder and more sustained. This has very important guiding significance for clinical personalized medicine.

## Discussion

The occurrence of acute hepatic injury is a complex interaction of the environment and heredity. Biologists have done a lot of research on this and have summarized a large part of the results in the NCBI-Gene database. In order to understand the molecular mechanism of the pathogenesis of acute liver injury. We analyzed acute liver injury-associated genes and clustered into 21 modules of biological functions and involved signal transduction pathways. Observing the modules involved in the function and pathway, we found that up to 16 modular genes were significantly involved in positive regulation of cell and cellular component movements. Last but not least, 13 modules were significantly involved in the regulation of systematic procedure and protein serine/threonine kinase activity. Protein serine/threonine kinases play a key role in the inflammatory response. For example, protein kinase D (PKD) inhibitors can inhibit the activation of mitogen activated protein kinase (MAPKs), thereby reducingthe acute liver injury induced by lipopolysaccharide^10^. The occurrence of inflammation and overactive immune response are important components of the development of acute liver injury.

In the search for module driving force, we have found that 1550 ncRNAs to drive modules. RNAi-mediated inhibition of MALAT1 can reduce myofibroblast markers and promote SIRT1 protein recovery, which regulates liver fibrosis process^11^. CRNDE has been reported to be abnormally high in hepatocellular carcinoma (HCC), which may promote lymph node metastasis (TNM) of liver cancer,thus it becomes targeted therapy of liver cancer^12,13^. On the other hand, miR-26b-5p has been confirmed to induce epithelial mesenchymal transition (EMT) by targeting the inhibition of SMAD1 and BMP4/Smad1 signals. Thereby, it can prevent the metastasis and proliferation of hepatocellular carcinoma and hepatocellular carcinoma (HCC)^14,15^. In addition, we explored 68 TFs giving priority to NFKB1, SP1 and EPAS1. The main function of NFKB1 is to participate in NF-κB signaling and is closely related to the body’s immunity, inflammatory response and liver regeneration^16–18^. At the same time, NFKB1 plays an important regulatory role in aging-related chronic liver diseases, and its gene promoter polymorphisms increase the susceptibility of hepatocellular carcinoma to a certain extent^19,20^. Subrata LS *et al*.’s work showed that Sp1 is involved in the response of interleukin (IL)-6 to the expression of lymphotoxin-β liver in tumor necrosis factor family members and affects liver regeneration associated with chronic liver injury^21^. Wang Y *et al*.’s work also confirmed that the sequence-specific transcription factor is involved in the regulation of monocyte chemoattractant protein-1 expression, which is of great significance in the treatment of endotoxin-mediated renal injury^22^. Finally, EPAS1 has been reported to involve in the regulation of decidual infiltration and portal vein invasion, playing an important role in the progression and prognosis of hepatocellular carcinoma (HCC)^23,24^. These key drivers play their respective regulatory roles and have an important impact on the formation of acute liver injury.

It is wellknown that drug therapy has always been plagued by drug resistance and drug toxicity. A comprehensive and systematic understanding of the molecular mechanisms underlying the pathogenesis of acute liver injury is the key to drug development and personalized treatment for acute liver injury. At the same time, the efficacy of bifendate and muaddil sapra for acute liver injury was analyzed and compared. STAT1 and IRF8 in muaddil sapra’s therapeutic related genes are also the pivot genes of the regulatory module. Among them, the JAK2/STAT1 signaling pathway plays an important role in the inflammatory response^25,26^. IRF8 is also involved in the regulation of inflammatory responses in a variety of cells^27,28^. In addition, PRIM2 and PIP5K1B in muaddil sapra treatment-related ncRNAs are also the pivot genes of the regulatory modules. PRIM2 may be involved in cell proliferation, thereby regulating traumatic diseases^29^. In addition, PIP5K1B has a negative regulatory effect on oxidative stress^30^. SNORD43 and RNU11 in the bifendate therapeutic gene also function as regulatory modules. Among them, RNU11 participates in many cellular processes through interaction with various RNAs^31^.

On the other hand, the interaction network was constructed by differentially expressed proteins regulated by two drugs and the degree of nodes was calculated. The higher the degree of the node, the more important it is considered to be. Bifendate can plays a major therapeutic role in the regulation of immunity and inflammation by regulating Rac2, Fermt3 and Plg as key molecular interaction network clusters. Bezerra JA *et al*.’s experiments have confirmed that plasminogen (Plg) can remove necrotic tissue and exert hepatic remodeling and regeneration from the diseased liver microenvironment during acute hepatic injury induced by carbon tetrachloride^32^. The muaddil sapra mainly activates Sqle and Stat1, Stat1 not only drives the modulesbut also participates in the adjustment of the modules for therapeutic purposes. From the pharmacokinetics perspective, bifendate primarily activates metabolic molecules such as Cyp3a11, Cyp4a10and Cyp4a14, rapidly regulating drug metabolism. They are members of the cytochrome P450 (CYP) superfamily and play a key role in drug metabolism^33–35^. On the other hand, muaddil sapra also targetsCyp1a2and Cyp3a11 and other metabolic molecules, but its activated metabolic molecules are less than bifendate, so it can be predicted that its drug metabolism is relatively slow, and its efficacy is more lasting.

In summary, we can conclude that the therapeutic efficacy of the two drugs: bifendate treatment process is fierce, causing the body to quickly respond to injury, effective, but its metabolism is also rapid. It has a significant effect on the treatment of emergency injuries. Muaddil sapra, on the other hand, is mild and persistent. In short, this project not only provides a basic theory for the further study of the pathogenesis of acute liver injury, but also offers a valuable reference for its clinical use and personalized treatment.

## Materials and Methods

### Proteome and transcriptome quantitation in model mice

Objects of the study are Kunming mice (half male and female, 18–22 g, Specific Pathogen Free) and the protocols for the experiments were approved by the Xinjiang Institute of Traditional Uyghur Medicine Ethics Committee on Animal Experimentation (China, production certificate no: SCXK2016-0001), and were conducted in accordance with China’s National Animal Health and Medical Research Council regulations for animal care and use for scientific purposes. All mice were housed in boxes and provided with drinking water and standard diets, monitored daily before the experiment. First, all mice were divided into four groups, one of which was treated with blanks and the remaining three were treated with carbon tetrachloride (acute liver injury model). Then three groups of acute liver injury mice were treated as blank treatment, bifendate treatment (30 mg/kg) and muaddil sapra treatment (16 g crude drug/kg). Among them, muaddil sapra is recorded in the Uyghur medical ancient book Mizani Tibi on page 533. It has the regulation of abnormal bile fluid, liver protection, heat-clearing and detoxification.The prescription components are Tianshan amaranth, sleeping lotus, chicory, and medicinal grass. Rose, senna, Able and other 7 herbs.Next, liver samples were removed, and RIPA strong lysate was added to 10 mg/80 μL. The tissue was cut with scissors, and the tissue was broken with a tissue disrupter and placed on ice for 15 min with high-speed centrifugation (12,000 g, 4 °C, 15 min). Finally, a series of transcriptome quantification and proteome quantification were performed, such as proteolysis, high pH reverse phase separation, nano-HPLC-MS/MS analysis, DIA data acquisition and analysis. The resulting data has been uploaded to the NCBI-GEO dataset.

### Co-expression analysis of acute liver injury-related gene expression profile

The National Center for Biotechnology Information (NCBI-Gene) database contains many published studies of acute liver injury-related studies. In order to systematically investigate the molecular mechanism of acute liver injury related genes, 715 related genes were obtained from the Gene database (GSE71379), and the interaction protein of with score >950 was sought in the human protein interaction database (String)^36^. Then, 370 samples of acute liver injury model mouse were downloaded from the NCBI-GEO plan database^37^, and a matrix of RNA expression profiles of genes associated with acute liver injury was constructed.

In order to explore the co-expression of 4541 acute liver injury-related genes, we used weighted gene co-expression network analysis (WGCNA)^38^ to analyze RNA expression matrix of acute liver injury-related genes to search for synergistically expressed gene modules. First, the correlation coefficients are used to calculate the correlation coefficient (Person Coefficient) between any two genes. The connection between genes in the network obeys scale-free networks, making the algorithm more biologically meaningful. Then, a hierarchical clustering tree is constructed by the correlation coefficient between genes, and different branches of the cluster tree represent different gene modules, and different colors represent different modules.

### Identification of ncRNAs and TF core drivers

The transcriptional and post-transcriptional regulation of genes is often driven by non-coding genes (ncRNAs) and transcription factors (TFs). In order to explore the driving forces of the co-expression module of genes involved in acute liver injury, we used the ncRNA-mRNA interaction pairs with score > = 0.5 in the RAID v2.0 database^39^ and all human TF interaction pairs in the TRRUST v2 database as a background set to perform pivot analysis. Pivot analysis refers to finding at least two interacting pairs of drivers in a target pair and calculating the significance of the driver-module interaction based on hypergeometric tests,with p value < 0.01.

### GO function and KEGG pathway enrichment

Exploring the function of genes and participating signal pathways is often an effective means to study the molecular mechanisms of diseases. Therefore, we used the R language Clusterprofile package^40^ for the 21 module genes to perform Go function (pvalueCutoff = 0.01, qvalueCutoff = 0.01) and KEGG pathway (pvalueCutoff = 0.05, qvalueCutoff = 0.2) enrichment analysis. In addition, we use the Cytoscape’s BinGO^41^ application to perform functional and path analysis of the integrated module network.

### Proteomics and transcriptome analysis

For proteomic data, we first used PEAKS DB^42^ for quality control. Then, we used Spectronaut Pulsar software^43^ for differential protein statistics, followed by R language TopGO package^44^ for functional and pathway enrichment analysis.

## Supplementary information

Dataset 1

Dataset 2

Dataset 3

Dataset 4

Dataset 5

Dataset 6

Dataset 7
